# Combination of PI3K and MEK inhibitors yields durable remission in PDX models of *PIK3CA*-mutated metaplastic breast cancers

**DOI:** 10.1186/s13045-020-0846-y

**Published:** 2020-02-22

**Authors:** F. Coussy, R. El Botty, M. Lavigne, C. Gu, L. Fuhrmann, A. Briaux, L. de Koning, A. Dahmani, E. Montaudon, L. Morisset, L. Huguet, L. Sourd, P. Painsec, S. Chateau-Joubert, T. Larcher, S. Vacher, S. Melaabi, A. Vincent Salomon, E. Marangoni, I. Bieche

**Affiliations:** 1 Unit of Pharmacogenomics, Department of Genetics, Institut Curie, Paris, France; 2 Laboratory of Preclinical Investigation, Department of Translational Research, Institut Curie Research Center, Paris, France; 3 Department of Medical Oncology, Institut Curie, Paris, France; 4 Department of Biopathology, Institut Curie, Paris, France; 5 Translational Research Department, RPPA Platform, Institut Curie Research Center, Paris, France; 6 BioPôle Alfort, National Veterinary School of Alfort, Maison Alfort, France; 7 INRA, APEX-PAnTher, Oniris, Nantes, France; 8 Inserm U1016, University Paris Descartes, Paris, France

**Keywords:** Metaplastic breast cancer, PI3K inhibitor, MEK inhibitor, Combination of targeted therapies

## Abstract

**Background:**

Metaplastic breast cancer (MBC) is a rare form of breast cancer characterized by an aggressive clinical presentation, with a poor response to standard chemotherapy. MBCs are typically triple-negative breast cancers (TNBCs), frequently with alterations to genes of the PI3K-AKT-mTOR and RTK-MAPK signaling pathways. The objective of this study was to determine the response to PI3K and MAPK pathway inhibitors in patient-derived xenografts (PDXs) of MBCs with targetable alterations.

**Methods:**

We compared survival between triple-negative MBCs and other histological subtypes, in a clinical cohort of 323 TNBC patients. PDX models were established from primary breast tumors classified as MBC. PI3K-AKT-mTOR and RTK-MAPK pathway alterations were detected by targeted next-generation sequencing (NGS) and analyses of copy number alterations. Activation of the PI3K-AKT-mTOR and RTK-MAPK signaling pathways was analyzed with reverse-phase protein arrays (RPPA). PDXs carrying an activating mutation of *PIK3CA* and genomic changes to the RTK-MAPK signaling pathways were treated with a combination consisting of a PI3K inhibitor and a MEK inhibitor.

**Results:**

In our clinical cohort, the patients with MBC had a worse prognosis than those with other histological subtypes. We established nine metaplastic TNBC PDXs. Three had a pathogenic mutation of *PIK3CA* and additional alterations to genes associated with RTK-MAPK signaling. The MBC PDXs expressed typical EMT and stem cell genes and were of the mesenchymal or mesenchymal stem-like TNBC subtypes. On histological analysis, MBC PDXs presented squamous or chondroid differentiation. RPPA analysis showed activation of the PI3K-AKT-mTOR and RTK-MAPK signaling pathways. In vivo, the combination of PI3K and MAPK inhibitors displayed marked antitumor activity in PDXs carrying genomic alterations of *PIK3CA*, *AKT1*, *BRAF*, and *FGFR4*.

**Conclusion:**

The treatment of metaplastic breast cancer PDXs by activation of the PI3K-AKT-mTOR and RTK-MAPK pathways at the genomic and protein levels with a combination of PI3K and MEK inhibitors resulted in tumor regression in mutated models and may therefore be of interest for therapeutic purposes.

## Introduction

Metaplastic breast carcinoma (MBC) is a rare, heterogeneous group of breast cancers characterized by differentiation of the neoplastic epithelium into squamous and/or mesenchymal elements, including spindle, chondroid, osseous, and rhabdomyoid cells. These tumors may consist entirely of metaplastic elements or be composed of a complex mixture of carcinoma and metaplastic areas [1, 2]. Most MBCs have a triple-negative phenotype, with no estrogen receptor (ER) or progesterone receptor (PR) expression and no overexpression of ERBB2 [2, 3]. The clinical presentation of MBC is characterized by a rapidly growing tumor mass at diagnosis, with a higher incidence of stage III and IV disease and a higher risk of local recurrence than for invasive ductal carcinomas [4, 5]. MBC is typically chemoresistant. Neoadjuvant chemotherapy and metastatic treatment are of limited efficacy for reducing tumor burden and preventing disease progression [6–8]. Survival is lower in MBC patients than in non-MBC patients [7, 9].

Gene expression analyses have shown that most MBCs are of the mesenchymal-like and mesenchymal stem-like molecular subtypes, according to the classification of triple-negative breast cancers [10].

The rarity of MBC has limited opportunities for characterizing the molecular genetic landscape in large cohorts of tumors, but several small case series have been analyzed, and molecular details are beginning to emerge [11]. Several studies have demonstrated a high frequency of phosphoinositide (PI)-3 kinase pathway aberrations, including frequent *PIK3CA* mutations and *TP53* mutations [12]. Other changes, such as *CDKN2A* loss and *EGFR* amplification, have been described, but at low frequency [12–14]. Krings et al. sequenced a panel of 28 MBCs and found strong enrichment in aberrations of the *PIK3CA/PIK3R1* (61%) and RAS-MAP kinase (25%) pathways, affecting *HRAS*, *KRAS*, and *NF1* in particular [15]. Similarly, McCart et al. performed whole-exome sequencing on 30 cases and found mutations of *TP53*, *PTEN*, and *PIK3CA* and an overrepresentation of *NF1* mutations [1].

Alterations to the PI3K-AKT-mTOR pathways are potentially promising targets for MBC management. However, no clinical data for PI3K inhibitor treatment have been reported for MBC patients, due to the rarity of these tumors, and preclinical data for MBC patient-derived xenografts are also lacking.

We report here the establishment and molecular characterization of MBC PDXs. We show, for the first time, that a combination of PI3K and MEK inhibitors is highly effective against MBC PDXs with *PIK3CA* mutations and alterations to the RTK-MAPK signaling pathway.

## Materials and methods

### Clinical cohort

Samples from 323 unilateral invasive triple-negative primary breast tumors excised from women managed at Institut Curie (Paris and Saint-Cloud, France) between 1980 and 2015 were analyzed (Additional file 1: Table S1). Most patients (67%) were diagnosed and treated after 2000. All patients admitted to our institution before 2007 were informed that their tumor samples might be used for scientific purposes and were given the opportunity to refuse such use. Since 2007, patients admitted to our institution also give express consent for the use of their samples for research purposes, by signing an informed consent form. Patients (mean age, 56 years; range, 28–91 years) met the following criteria: primary unilateral non-metastatic TNBC, with full clinical, histological, and biological data and full follow-up at Institut Curie. Median follow-up was 7.8 years (range 8 months to 36 years). Seventy-eight patients had developed metastases within 10 years.

### Patient-derived xenografts

PDXs were established from the engraftment of primary breast tumors with a procedure described elsewhere [16–18]. Female Swiss nude mice were purchased from Charles River Laboratories and maintained under specific pathogen-free conditions. The experimental protocol and animal housing were in accordance with institutional guidelines and with the recommendations of the French Ethics Committee (Agreement B75-05-18, France). Three metaplastic TNBC PDXs with genomic alterations were chosen for experimental analysis: HBCx-60, HBCx-165, and HBCx-178. BYL-719 (PI3K inhibitor) and selumetinib (MEK inhibitor) were purchased from Medchem Express. BYL-719 was administered five times per week, at doses of 35 mg/kg, by oral gavage. Selumetinib (MEK inhibitor) was administered five times per week, at doses of 100 mg/kg (50 mg/kg, bid), by oral gavage. Adriamycin (DOX, doxorubicin, Teva Pharmaceuticals) and cyclophosphamide (Endoxan, Baxter) were administered by the intraperitoneal (i.p.) route, at doses of 2 and 100 mg/kg, respectively, every 3 weeks. We included 10 mice per groups. Tumor growth was evaluated by measuring two perpendicular tumor diameters with calipers twice weekly. Individual tumor volumes were calculated as follows: *V* = (*a* × *b*)^2^/2, where “*a*” is the largest diameter, and “*b*” is the smallest diameter. For each tumor, volumes are expressed relative to the initial volume, as a relative tumor volume (RTV). Tumor growth inhibition (TGI) was assessed by dividing mean RTV (relative tumor volume) in the treated group by mean RTV in the control group at the same time. The statistical significance of TGI was assessed by comparing tumor volumes between the treated and control groups in paired Student’s *t* tests. Stable disease was defined as the percentage change in volume, between 0 and − 50.

### Transcriptomic data analysis

We used gene expression arrays for the transcriptomic profiling of 64 PDX TNBCs. The concentration and integrity/purity of each RNA sample were assessed with the RNA 6000 LabChip kit (Agilent) and an Agilent 2100 bioanalyzer. GeneChip Human 1.1 ST arrays were hybridized according to Affymetrix recommendations, with the WT Expression Kit protocol (Life Technologies) and Affymetrix labeling and hybridization kits. Arrays were normalized according to the RMA normalization procedure, with the oligo package [19]. No additional human-mouse cross-hybridization filtering was applied, as our xenograft samples contained less than 5% mouse cells (percentage determined by RT-PCR quantifying transcripts of the ubiquitously expressed *TBP* gene with specific mouse and human primers pairs), a percentage too low to affect the expression profiles obtained with HuGene1.0 arrays [20]. The TNBC molecular subtypes of the PDXs were determined from gene expression data, with the TNBCtype software developed by Chen et al [21].

### Somatic mutation analysis

We analyzed 64 PDXs by the targeted NGS of 95 genes, chosen from the genes most frequently mutated in breast cancer (> 1%) and including potential therapeutic targets, as previously described [22]. Briefly, NGS was performed on an Illumina HiSeq 2500 sequencer, and the genomic variants were annotated with the COSMIC and 1000 genome databases. Variants with a low allelic frequency (< 5%) or low coverage (< 100x) were excluded from the analysis. Deleterious genomic alterations were defined as follows: (i) for oncogenes, only gain-of-function mutations were considered (i.e., hotspot missense mutations, in-frame insertions/deletions/splicing reported to be oncogenic), and (ii) for tumor suppressor genes (TSG), only loss-of-function mutations were considered (i.e., biallelic truncating alterations (nonsense mutations, frameshift insertions/deletions/splicing) or monoallelic truncating alterations associated with heterozygous deletion detected by copy number analysis). Genomic variants were biologically validated by comparison with the COSMIC, TumorPortal, and cBioportal databases [7, 15].

### Somatic copy number alteration (SCNA) analysis

PDXs were profiled with Affymetrix genomics arrays: 24 with SNP 6.0 and 37 with the Cytoscan HD array. Genome-wide copy number analysis was performed with Affymetrix SNP arrays, as previously described [23, 24]. SNP 6.0 or Cytoscan HD arrays were used with 500 ng and 250 ng of gDNA, respectively, as the input material, as recommended by the manufacturer. Raw data were normalized with Genotyping console (SNP6.0 arrays) or Chromosome Analysis Suite (Cytoscan HD arrays). The focal amplification of oncogenes was defined as a log ratio > 1.58 (6 copies per diploid genome) and a maximum size of < 10 Mb. The biallelic inactivation of TSGs was defined as homozygous deletion or truncating mutations associated with heterozygous deletion. Copy number alterations were compared with cBioPortal data for TCGA breast cancer [25, 26]. All PDX copy numbers are represented by the Circular Binary Segmentation algorithm [27], as implemented in the DNAcopy package for R, with a minimum width of 3, an alpha risk of 1%, and up to 10,000 permutations. Downstream analysis of the sample population was performed with GISTIC2.0 [28], with default settings. CGH explorer and CGHcall were used for visual representation of the results and figures [29]. The BRCAness signature consisted of large-scale state transitions (LST), defined as chromosomal breaks between adjacent regions of at least 10 Mb initially described by Popova et al. with Gap methodology [30].

### RT-qPCR in PDXs

Total RNA extraction and RT-qPCR have been described elsewhere [31]. The *TBP* gene (GenBank accession no. NM_003194) encoding the TATA box-binding protein (a component of the DNA-binding protein complex TFIID) was quantified as an endogenous RNA control, and each sample was normalized on the basis of its TBP content [28]. *N*-fold differences in target gene expression relative to the TBP gene (“Ntarget”), were determined as Ntarget = 2^ΔCtsample^, where the ΔCt value of the sample was obtained by subtracting the mean Ct value of the target gene from that of the TBP gene [32].

For the gene expression study in PDXs, mRNA levels were normalized to obtain a “basal mRNA level” (smallest amount of mRNA quantifiable (Ct = 35)) equal to 1. We analyzed the expression of genes involved in epithelial-mesenchymal transition (EMT) (*SNAI2*, *VIM*, *ACTA2*, *SPARC*, *TCF7L2*, *CAV1*) in all TNBC PDXs and included 49 with well-known transcriptomic classifications in the final analysis.

### Reverse-phase protein array (RPPA)

RPPA was performed as previously described [33] for 48 of the 64 TNBC PDXs (16 recent PDXs were not included in the RPPA analysis). We assessed pathway activation, by calculating a PI3K-AKT-mTOR and RTK-MAPK pathway score with normalized data. This score was obtained by calculating the sum of the protein levels for positive components and subtracting that for the negative components of the pathway (especially for PI3K-AKT-mTOR pathway): (i) PI3K p110 subunit β, P-AKT1 (Ser473), P-AKT1 (Thr308), P-4E-BP1, P-p70 S6 kinase, P-S6 ribosomal protein minus PTEN (Cell Signaling Technology®), and (ii) P-RSK2 (Cell Signaling Technology®).

### Immunohistochemistry (IHC)

Tumors from patients and xenografts were fixed in 10% neutral-buffered formalin, embedded in paraffin, and stained with hematoxylin and eosin. The same histologist (ML) compared morphology between patient and PDX tumors. Four representative MBCs with chondroid and squamous characteristics were compared (HBCx-130, HBCx-162, HBCx-165, HBCx-178).

### Western blot analysis

Proteins were extracted from tumors using a Laemmli buffer (50 mM Tris HCL pH 8, 2 mM DTT, 2% SDS, 5% glycerol), supplemented with protease and phosphatase inhibitors. Lysates were resolved on 10% agarose gels, transferred onto nitrocellulose membranes (Bio-Rad, Hercules, CA, USA), and immunoblotted with rabbit antibodies against AKT, p-AKT (ser 473), S6, p-S6 (Ser235/236), ERK, p-ERK (Thr202/Tyr204), MEK, p-MEK (Ser217/221), and GAPDH (Cell Signaling®). After washes, membranes were incubated with the appropriate horseradish peroxidase-conjugated affinity-purified goat anti-rabbit secondary antibodies (Jackson ImmunoResearch Laboratories, Inc., Interchim).

### Screening for *PIK3CA* mutations in patients

Hotspot *PIK3CA* mutations (exons 1, 2, 9, 20) were detected by sequencing cDNA fragments obtained by RT-PCR amplification. The exons of the gene to be screened were chosen on the basis of the mutation frequency reported in COSMIC: Catalogue of Somatic Mutations in Cancer (cancer.sanger.ac.uk/). Screening was performed by high-resolution melting curve analysis on a LightCycler 480 (Roche Diagnostics, Penzberg, Germany), with LCGreen Plus + Melting Dye fluorescence (Biotech, Idaho Technology Inc., Salt Lake City, UT). Details of the primers and PCR conditions used are available on request. The amplified products were sequenced with the BigDye Terminator kit on an ABI Prism 3130 automatic DNA sequencer (Applied Biosystems, Courtaboeuf, France) with a detection sensitivity of 5% mutated cells, and the sequences obtained were compared with the corresponding reference cDNA sequences (PIK3CA NM_006218). All mutations detected were confirmed in a second independent sequencing run.

### Statistical analysis

We compared the RT-qPCR values obtained for the MSL and M subtypes with those of other subtypes, in *t* tests. The proportions of genomic alterations between metaplastic and other types of PDX were compared in Fisher’s exact tests.

Metastasis-free survival (MFS) was assessed by determining the interval between diagnosis and the detection of the first distant metastasis. Overall survival (OS) was determined as the interval between diagnosis and death. Survival was estimated by the Kaplan-Meier method.

## Results

### Metaplastic breast cancer patients have a worse prognosis than patients with other histological subtypes

**Fig. 1:**
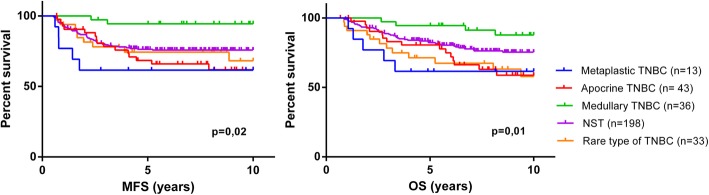
Metastasis-free survival (MFS) and overall survival (OS) in 323 different subtypes of TNBC (198 of no special type, 43 apocrine, 36 medullary, 13 metaplastic, and 33 rare TNBCs) analyzed in log-rank tests

We compared survival between triple-negative MBC and other histological subtypes in a clinical cohort of 323 TNBC patients treated at our institute, with a long follow-up. This cohort included 13 MBCs (4%), 43 apocrine BCs (13.3%), 36 medullary BCs (11.1%), 198 breast cancers of no special type (NST) (61.4%), and other rare forms of TNBC (10.2%) (oncocytic, acinic, lobular, adenoid cystic papillary, micropapillary, or mucinous). MBC patients had a worse prognosis than patients with other subtypes, in terms of metastasis-free survival (MFS) and overall survival (OS) (*p* = 0.02 and *p* = 0.01, respectively) (Fig. 1). The medullary TNBC subgroup had a particularly good prognosis.

We investigated the frequency of tumors with *PIK3CA* mutations with Sanger sequencing of *PIK3CA*. In the whole cohort of tumors, *PIK3CA* mutations were present in 15% of metaplastic tumors and 12.2% of the tumors of other subtypes (*p* = 0.66) (NST, 13%; apocrine, 14%; rare, 12%; medullary, 0%). *PIK3CA* mutations were of no prognostic values in the metaplastic subtype (data not shown).

### PDX models of metaplastic breast cancer are characterized by a mesenchymal phenotype

**Fig. 2:**
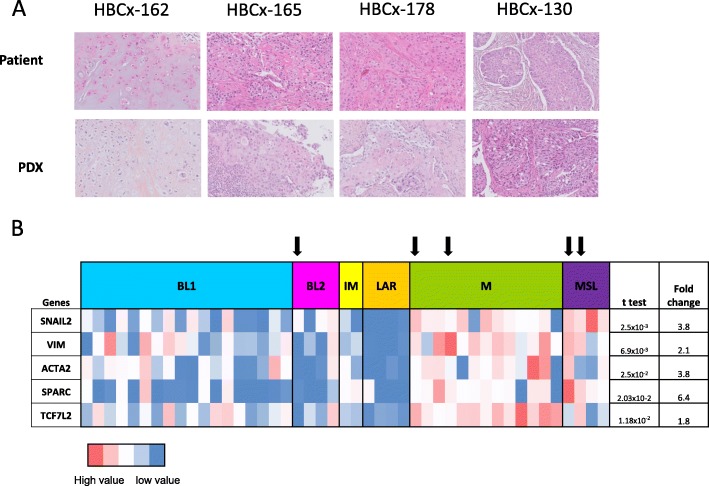
**a** Morphological comparison of four patients and PDX tumors (representative hematoxylin-eosin (H&E)-stained sections, magnification × 200). HBCx-162: metaplastic carcinoma with abundant chondromyxoid matrix in both patients and PDX tumors; HBCx-165, HBCx-178, HBCx-130: squamous cell metaplastic carcinoma, in both patients and PDXs. **b** RT-PCR analysis of the expression of EMT genes (*SNAIL2*, *VIM*, *ACTA2*, *SPARC*, *TCF7L2*) in TNBC PDXs (*n* = 48). Fold changes and *p* values are calculated for the comparison of gene expression in MSL and M PDXs with that in other TNBC subtypes. Arrow down symbol indicates metaplastic type

In our cohort of 64TNBC PDX models (the description of the first 61 PDX were recently published [22]), nine displayed metaplastic differentiation (14%) (Table 1). Three displayed fusiform differentiation, three had squamous characteristics, and three had mixed components (chondroid–fusiform or squamous–fusiform). Figure 2a shows the morphological features of four MBCs (patients and PDXs): HBCx-162, HBCx-130, HBCx-165, and HBCx-178. HBCx-162 was characterized by an abundant chondromyxoid matrix, and HBCx-130, HBCx-165, and HBCx-178 were squamous cell MBCs. The specific morphological characteristics of the initial tumors were also present in the corresponding PDXs. Squamous cell metaplastic carcinomas have a particular set of characteristics: tumor masses bordering cystic cavities, high degrees of nuclear pleomorphism, large polygonal cells with abundant eosinophilic cytoplasm, keratinization beads sometimes present, and abundant inflammatory infiltrate. These highly specific aspects are found in both PDXs and patient tumors. A similar phenomenon is observed for metaplastic carcinomas with mesenchymal chondroid differentiation: each pair of tumors displays an abundant cartilaginous and myxoid matrix enclosing the carcinomatous cells.

**Table 1:** Clinical and genomic characteristics of the metaplastic PDX models

ID	Type of graft	Age at diagnosis	BRCA mutational status	T	N	M	Breast surgery	Node surgery	Histologic type	Lymphovascular invasion	SBR grade	Chemotherapy (CT)	Type of CT	RT	Local relapse	Distant relapse	MFS (months)	OS (months)
HBCx-66	Residual disease post NCA	38	BRCA1	2	0	0	Mastectomy	Lymphadenectomy	Fusiform	No	3	Primary	Sequential	Yes	0	0	69	69
HBCx-90	Residual disease post NCA	77	No mutation	4d	0	0	Mastectomy	No	Fusiform	Yes	2	Primary	Taxane-based	Yes	1	1	11.5	11.5
HBCx-130	Residual disease post NCA	73	No mutation	2	0	0	Mastectomy	Sentinel lymph node biopsy	Squamous	No	3	Primary	Sequential	Yes	1	1	19	22
HBCx-162	Residual disease post NCA	48	No mutation	2	0	0	Tumorectomy	Sentinel lymph node biopsy	Chondroid and fusiform	No	3	Primary	Sequential	Yes	0	0	14	14
HBCx-165	Primary breast cancer	89	No mutation	2	0	0	Tumorectomy	Sentinel lymph node biopsy	Squamous	No	3	Adjuvant	Capecitabine	Yes	0	1	12	14
HBCx-178	Residual disease post NCA	61	No mutation	2	0	0	Tumorectomy	Sentinel lymph node biopsy	Squamous	No	3	Primary	Sequential	Yes	0	0	9	9
HBCx-23	Primary breast cancer	41	No mutation	1	0	0	Tumorectomy	Lymphadenectomy	Chondroid and fusiform	Yes	3	Adjuvant	Anthracyclin-based	Yes	0	0	83	83
HBCx-60	Primary breast cancer	30	No mutation	3	1	0	Mastectomy	Lymphadenectomy	Fusiform	No	3	Adjuvant	Sequential	Yes	0	1	103	106
HBCx-70	Residual disease post NCA	51	No mutation	2	0	0	Mastectomy	Lymphadenectomy	Squamous and chondroid	No	3	Primary	Sequential	Yes	0	1	15	27

*TNM* tumor, node, metastasis classification, *SBR* Scarff Bloom and Richardson, *CT* chemotherapy, *RT* radiotherapy, *MFS* metastasis-free survival, *OS* overall survival

Nine MBC PDXs were characterized at the transcriptomic level: four were classified as M (mesenchymal) or MSL (mesenchymal stem-like), 1 as BL2 (basal-like 2), and 3 were unstable. For further characterization of the phenotype of MBC PDXs, we performed a RT-PCR analysis of various genes involved in the epithelial-mesenchymal transition (EMT), a cancer cell phenotype previously associated with MBC [34], in 48 TNBC PDXs. We generated an expression heatmap for the *SNAI2*, *ACTA2*, *VIM*, *SPARC*, and *TCF7L2* genes in the various TNBC transcriptomic subtypes (14 unstable and 5 NA tumors were excluded from the analysis) (Fig. 2b). The expression of the *SNAI2*, *ACTA2*, *VIM*, *SPARC*, and *TCF7L2* genes was significantly stronger in the MSL/M subtypes than in the other subtypes (unpaired *t* test).

### Activation of the PI3K-AKT-mTOR and RTK-MAPK pathways in MBC PDX

The genomic alterations (mutations and copy number alterations) found in MBC PDXs concerned the PI3K and RTK-MAPK pathways.

**Fig. 3:**
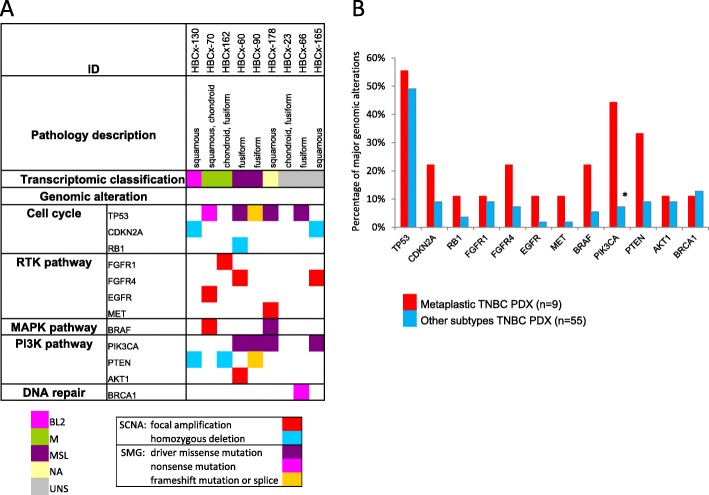
Genomic alterations in metaplastic PDXs and comparison with other TNBC subtypes. **a** Major genomic alterations (mutations and copy number alterations) in nine metaplastic PDXs with histological characteristics and transcriptomic classification. **b** Percentage of major genomic alterations in metaplastic TNBC PDXs versus the other histologic subtypes; *p = 0.01, Fisher’s test

Figure 3a shows the major genomic alterations in metaplastic PDX. Eight genetic alterations affecting the PI3K-AKT-mTOR signaling pathway were identified in six of the nine metaplastic PDX, including four *PIK3CA* mutations (44.4%), three *PTEN* genetic alterations (33.3%), and one *AKT1* amplification (11.1%). *PIK3CA* (activating mutations) was significantly more frequently altered in metaplastic TNCB than in other subtypes (Fisher’s test, *p* = 0.01) (Fig. 3b). Five of the nine metaplastic PDX (55%) harbored one alteration to the *TP53* gene (50% for other subtypes). The additional genetic alterations of theranostic interest were two alterations to the *BRAF* gene (one mutation and one focal amplification), two focal amplifications of *FGFR4*, and one focal amplification of *FGFR1*, *EGFR*, and *MET*. Four of the nine (44%) characterized MBCs displayed genomic changes to both the PI3K-AKT-mTOR and RTK-MAPK pathways (versus 11/55 for other subtypes, 20%).

We confirmed the activation of the PI3K-AKT-mTOR and RTK-MAPK signaling pathways at the protein level, by analyzing the expression of the major effectors of the PI3K-AKT-mTOR pathway (PI3-kinase, p-AKT, p-4E-BP1, p70-S6-kinase, p-S6RP, PTEN) and the RTK-MAPK pathway (p-MEK1, p-RSK2) by RPPA analysis. This analysis confirmed the activation of the PI3K pathway in metaplastic BCs. RSK2 is an effector of the MAPK pathway. Most metaplastic BCs had high RSK2 scores. However, by contrast to the PI3K pathway, the results of genomic and protein analysis were not well correlated (Additional file 2: Figure S1).

### *PIK3CA*-mutated PDXs respond to the combination of PIK3CA and MEK inhibitors

Based on the high frequency of concomitant alterations of the PI3K-AKT-mTOR and RTK-MAPK pathways, we hypothesized that the combination of PI3K and MEK inhibitors might constitute an efficient treatment strategy for MBC PDX. The PI3K inhibitor BYL-719 (alpelisib) and the MEK inhibitor selumetinib were tested in monotherapy and in combination in the HBCx-60 PDX (*FGFR4* amplification, *PIK3CA* mutation, and *AKT1* amplification), HBCx-165 PDX (*FGFR4* amplification, *PIK3CA* mutation) and HBCx-178(*BRAF* mutation and *PIK3CA* mutation) models. Two of these models are chemoresistant to anthracycline (HBCx-165 and HBCx-178).

**Fig. 4:**
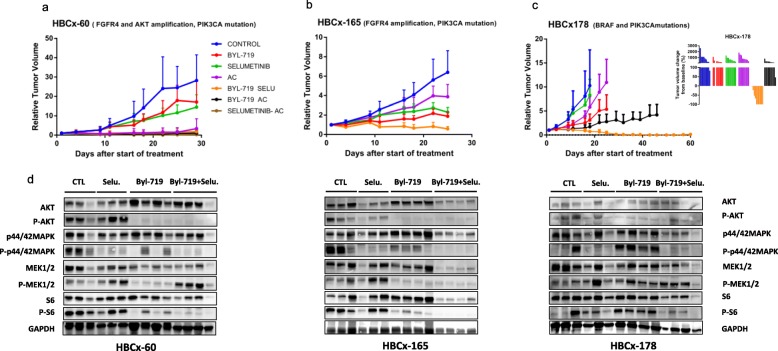
In vivo response to targeted therapies and chemotherapy in metaplastic PDXs. **a**, **b** Relative tumor growth (RTV) in HBCx-60 and HBCx-165 PDX models treated with BYL-719 (PI3KCa inhibitor) monotherapy, selumetinib (MEK inhibitor) monotherapy, chemotherapy with adriamycin and cyclophosphamide (AC), a combination of BYL-719 + selumetinib, BYL-719 + AC, and selumetinib + AC. Mean ± SD, *N* = 10 xenografts/group. **c** Relative tumor growth in the HBCx-178 PDX treated with BYL-719 (PI3KCa inhibitor) monotherapy, selumetinib (MEK inhibitor) monotherapy, chemotherapy with AC, or the combination of BYL-719 + selumetinib. **d** Western blot analysis of AKT, p-AKT (ser 473), S6, p-S6 (Ser235/236), ERK, p-ERK (Thr202/Tyr204), MEK, p-MEK (Ser217/221), and GAPDH in treated tumors of HBCx-60, HBCx-165, and HBCx-178. *N* = 3 − 4

In the HBCx-60 model, treatment with BYL-719 and selumetinib in monotherapy did not significantly decrease tumor growth (Fig. 4a). By contrast, the combination inhibited tumor growth, with a TGI of 94% relative to the control (*p* = 0.0018, Mann-Whitney test). In this chemosensitive model, the combination of targeted therapies induced a response similar to that obtained with chemotherapy. In the HBCx-165 model (Fig. 4b), treatment with BYL-719 and selumetinib in monotherapy reduced tumor growth, with a TGI of 74% (*p* = 0.028) and 66% (*p* = 0.16), respectively, but xenografts showed no tumor growth arrest or regression. Conversely, in the group of xenografts treated with the combination of BYL-719 and selumetinib (TGI of 91%), six mice had stable disease, and two xenografts displayed tumor regression (data not shown). The combination was superior to BYL-719 alone (*p* = 0.0012). Finally, in the HBCx-178 PDX model, treatment with BYL-719 resulted in a TGI of 70% with no tumor regression, whereas the combination of BYL-719 and selumetinib resulted in a complete response in six of ten mice (Fig. 4c).

Monotherapies induced no significant change, but the combination was highly effective, with significant tumor regression observed in all models and a complete response frequency of 60% for HBCx-178.

To assess inhibition of PIK3 and MAPK signaling pathways, we analyzed the phosphorylation status of AKT, S6, ERK, and MEK in treated tumors harvested at the end of treatment by western blot (Fig. 4d). P-AKT was strongly inhibited in xenografts treated by BYL-719 alone or associated with selumetinib in HBCx-60 and HBCx-165 PDX and, to a lesser extent, in HBCx-178. P-S6 was strongly inhibited in xenografts of the combination arm. In the 3 PDX models, P-ERK was inhibited in selumetinib-treated xenografts and strongly inhibited in xenografts treated with the combination BYL-719 + selumetinib. In the two models (HBCx-60 and HBCx-165), P-MEK was inhibited in BYL-719 treated tumors. Overall, these results show strong inhibition of both PIK3 and MAPK signaling pathways in tumors treated by BYL-719 associated with selumetinib.

## Discussion

Metaplastic breast carcinoma constitutes a group of histologically and molecularly diverse tumors. MBC is rare, but patients with metastatic MBC have been shown to have lower rates of response and poorer survival [35] than patients with other subtypes and to display early relapse. Optimal treatment strategies for metastatic MBC based on genomic analysis are urgently required.

Our results confirm that (i) metaplastic breast carcinomas are mostly of the mesenchymal-like and mesenchymal stem-like TNBC subtype, according to Lehmann’s classification [3], and (ii) these subtypes overexpress EMT genes. Like TNBC-NST, MBCs frequently harbor somatic *TP53* mutations [13]. By contrast, MBCs more frequently display mutations affecting the PI3K-AKT-mTOR pathway [11, 12, 36]. We found that 66% of our metaplastic PDXs displayed mutations of the PI3K-AKT-mTOR pathway; a higher proportion presented PIK3CA mutations and genomic alterations affecting both the PI3K-AKT-mTOR and RTK-MAPK pathways which occurred in 44% of our MBCs. This association was reported in a previous study by Krings et al. [15]. Finally, our findings confirm the activation, at the protein level, of the PI3K-AKT-mTOR and, to a lesser extent, the RTK-MAPK pathway in our metaplastic PDXs.

In three metaplastic PDX models with genomic alterations to both the PI3K-AKT-mTOR and RTK-MAPK pathways, we tested a PI3K inhibitor, a MEK inhibitor, their combination, and their association with an anthracycline (usual chemotherapy in breast cancer). BYL-719 is an effective PI3K inhibitor because, unlike previous generations of PI3K inhibitors, its specificity for the p110 unit may be more clinically relevant and less toxic than pan-PI3K inhibition. The American Food and Drug Administration recently approved this treatment in combination with fulvestrant in metastatic settings for hormone receptor-positive, *PIK3CA*-mutated breast cancer. Indeed, association with BYL-719 increased median progression-free survival from 5.7 to 11 months. PI3K inhibitors are of potential clinical interest for the treatment of metaplastic BC, as suggested by a case report from the BELLE 4 clinical trial in which a durable response obtained in a patient with metaplastic BC following treatment with a combination of buparlisib (PI3K inhibitor) and paclitaxel [37]. Nevertheless, the results for the total population were disappointing.

In two models (with *PIK3Ca* mutation and *FGFR4* amplification), monotherapies decreased tumor growth without inducing regression. This insufficient efficacy can be explained by (i) the co-occurrence of two activating pathways. Indeed, the co-occurrence of targetable alterations has already been described in TCGA [38] and was found in 44% of our MBC PDXs, (ii) the presence of *AKT1* mutation in one model, or (iii) crosstalk and compensatory mechanisms between the PI3K-AKT-mTOR and RTK-MAPK pathways [39]. By contrast, the combination of PI3K and MEK inhibitors induced some complete and durable responses in all three PDX models that were associated to strong inhibition of both PI3K and MAPK signaling pathways. In particular, in the HBCx-178 model (*BRAF* and *PIK3CA* mutation), 44% of mice presented a durable complete response. Comparison with chemotherapy highlighted (i) the superiority of the combination of two targeted therapies in chemoresistant models (HBCx165 and HBCx-178) and (ii) the similar efficacy of these two treatments in a chemosensitive model (HBCx-60).

## Conclusions

Our analysis highlights the co-occurrence of actionable alterations and opportunities for combination treatment in metaplastic breast cancer. In clinical practice, the combination of specific PI3K and MEK inhibitor treatments seems to be feasible, with a manageable safety and toxicity profile (NCT01449058). A second phase I trial (NCT01449058) is currently underway, to test the safety and tolerability of BYL-719 and trametinib (MEK inhibitor). Our results provide a rationale for the genomic selection of MBCs for investigations of the use of a PI3K inhibitor in combination with a MEK inhibitor.

This work was funded by Institut Carnot and SIRIC2 (INCa-DGOS-Inserm_12554) grants.

## Supplementary information

**Additional file 1: Table S1.** Characteristics of TNBC patients.

**Additional file 2: Figure S1.** Genomic and proteic analysis of PI3K-AKT-mTOR and RTK-MAPK pathways in PDX models.

## Data Availability

The datasets used and/or analyzed during the current study are available from the corresponding author on reasonable request.

## Abbreviations

ER: Estrogen receptor
MBC: Metaplastic breast cancer
NA: Not available
NST: No special type
PDX: Patient-derived xenografts
PR: Progesterone receptor
RPPA: Reverse-phase protein arrays
RTV: Relative tumor volume
TGI: Tumor growth inhibition
TNBC: Triple-negative breast cancer
TSG: Tumor suppressor genes
