# Supplementary material for: Combination of PI3K and MEK inhibitors yields durable remission in PDX models of PIK3CA-mutated metaplastic breast cancers
Source: J Hematol Oncol. 2020 Feb 22;13:13. doi: 10.1186/s13045-020-0846-y (PMC7036180; doi:10.1186/s13045-020-0846-y)
Supplement: Supplementary file 1 — Additional file 1: Table S1. Characteristics of TNBC patients. [file 13045_2020_846_MOESM1_ESM.docx]

| **Age** (mean) y (min-max) | | 56 (28-91) |
| --- | --- | --- |
|  |  | *N* (%) |
| **T (TNM)** | 1 | 115 (35.6) |
|  | 2 | 194 (60) |
|  | 3 | 14 (4.4) |
| **N (TNM)** | 0 | 200 (62) |
|  | 1 | 79 (25) |
|  | 2 | 30 (9) |
|  | 3 | 12 (4) |
| **SBR** | 1 | 5 (1.6) |
|  | 2 | 41 (13) |
|  | 3 | 270 (85.4) |
| **Chemotherapy** | Yes | 244 (76.3) |
|  | No | 76 (23.7) |
| **Radiotherapy** | Yes | 242 (83.2) |
|  | No | 49 (16.8) |
| **Subtype** | NST | 198(61.4) |
|  | Medullary | 36 (11.1) |
|  | Rare | 33 (10.2) |
|  | Apocrine | 43 (13.3) |
|  | Metaplastic | 13 (4) |

Table S1: Characteristics of TNBC patients
